# Fucoidan extract enhances the anti-cancer activity of chemotherapeutic agents in breast cancer cells

**DOI:** 10.1186/1753-6561-7-S6-P70

**Published:** 2013-12-04

**Authors:** Sanetaka Shirahata, Zhonguan Zhang, Toshihiro Yoshida, Hiroshi Eto, Kiichiro Teruya

**Affiliations:** 1 Department of Bioscience and Biotechnology, Faculty of Agriculture, Kyushu University, Fukuoka 812-8581, Japan; 2 Yosida Clinic, Osaka 532-0002, Japan; 3 Daiichi Sangyo Co. Ltd., Osaka 530-0037, Japan

## Background

Fucoidan, a fucose-rich polysaccharide isolated from brown alga, is currently under investigation as a new anti-cancer compound [1-4]. In the present study, fucoidan extract (FE) from *Cladosiphon navae-caledoniae *Kylin was prepared by enzymatic digestion. We investigated whether a combination of FE with chemotherapeutic agents had the potential to improve the therapeutic efficacy of cancer treatment.

## Materials and methods

Estrogen receptor (ER)-positive MCF-7 and ER-negative MDA-MB-231 breast cancer cells were cultured in DME medium supplemented with 10% fetal bovine serum in a humidified atmosphere of 5% CO_2 _at 37 °C. The abalone glycosidase-digested fucoidan extract (FE) was obtained from Daiichi Sangyo Corporation (Osaka, Japan). The cells were treated with FE and chemotherapeutic agents like cisplatin, tamoxifen or paclitaxel. The cell growth was determined by MTT assay. Apoptosis was evaluated using annexin V binding assay and flow cytometry analysis. Signaling proteins were analyzed by western blot. Intracellular reactive oxygen species (ROS) were determined using DCFH-DA and determined using IN Cell Analyzer 1000. The reduced glutathione (GSH) concentration was measured by the GSH assay kit.

## Results

**Figure 1:**
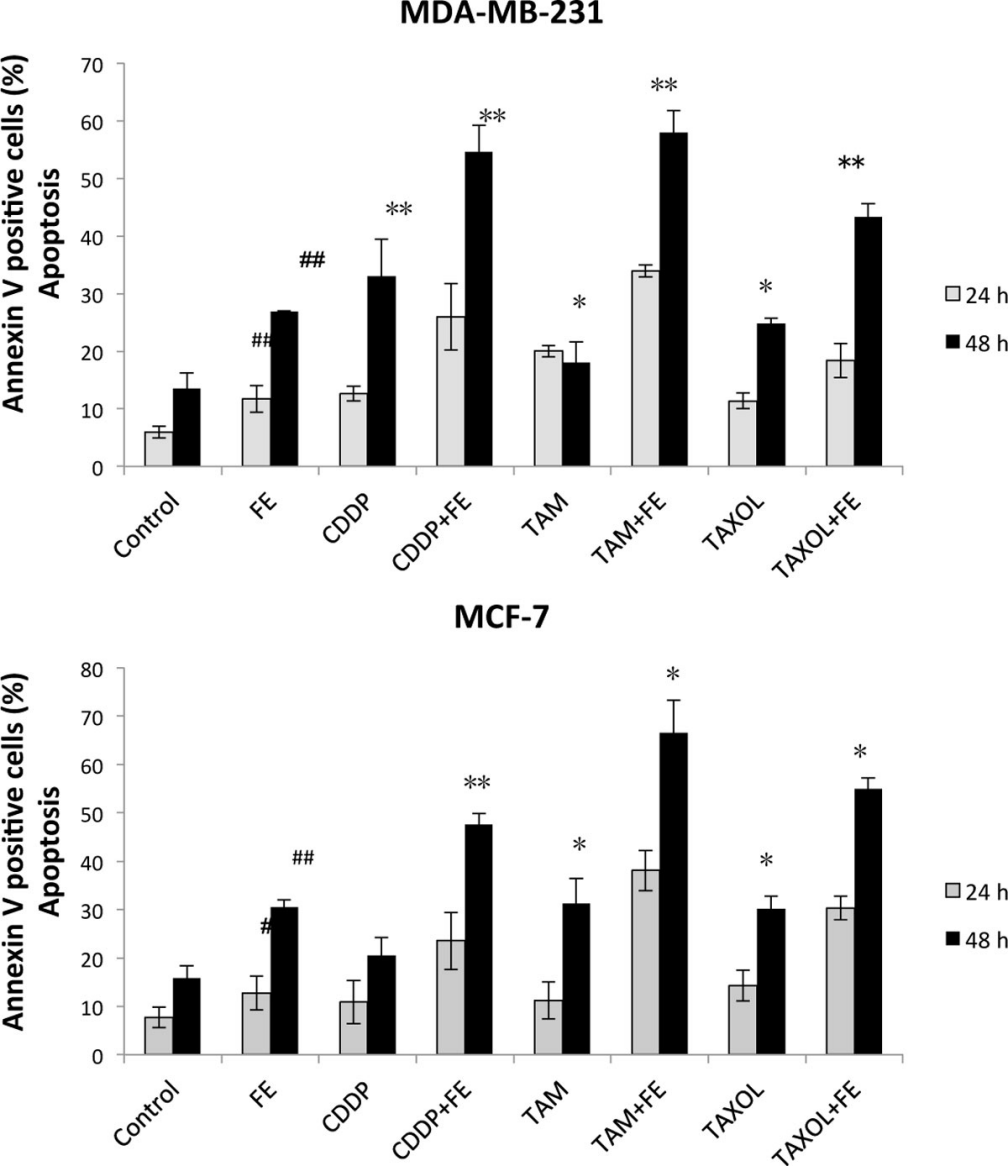
**Synergistic induction of apoptosis by co-treatment Analysis of apoptotic cells by annexin/PI double-staining using the IN Cell Analyzer 1000**. MDA-MB-231 and MCF-7 cells were treated for different times with 200 μg/mL FE alone or 200 μg/mL FE in combination with 5 μM CDDP, 10 μM TAM or 2.5 nM TAXOL after 48 h of treatment. All results were obtained from three independent experiments. A significant difference from control is indicated by *p *< 0.05 (#) or *p *< 0.01 (##); a significant difference from single treatments is indicated by *p *< 0.05 (*) or *p *< 0.01 (**).

The co-treatments significantly induced cell growth inhibition, apoptosis, as well as cell cycle modifications in MDA-MB-231 and MCF-7 cells. FE enhanced apoptosis in cancer cells that responded to treatment with cisplatin, tamoxifen, or paclitaxel after 48 h of treatment (Figure 1). FE enhanced the downregulation of the anti-apoptotic proteins Bcl-xL and Mcl-1 by these chemotherapeutic drugs. The combination treatments led to an obvious decrease in the phosphorylation of ERK and Akt in MDA-MB-231 cells, but increased the phosphorylation of ERK in MCF-7 cells. In addition, we observed that combination treatments enhanced intracellular ROS levels and reduced glutathione (GSH) levels in breast cancer cells, suggesting that induction of oxidative stress was an important event in the cell death induced by the combination treatments.

FE protected normal human fibroblast TIG-1 cells from apoptosis by cisplatin and tamoxifen, suggesting its favorable characteristic for application to cancer therapy.

## Conclusions

• Combination of FE and three chemotherapeutic agents exhibit highly synergistic inhibitory effects on the growth of breast cancer cells.

• Combination treatments induced modifications in cell cycle distribution.

• Combination treatments modified the Bcl-2 expression, and ERK and Akt phosphorylation induced by FE, demonstrating different effects on apoptotic pathways in MDA-MB-231 cells and MCF-7 cells.

• Generation of intracellular ROS and depletion of GSH are related to the cell death in combination treated -breast cancer cells.
